# Design of a Papain Immobilized Antimicrobial Food Package with Curcumin as a Crosslinker

**DOI:** 10.1371/journal.pone.0121665

**Published:** 2015-04-23

**Authors:** Cynthya Maria Manohar, Veluchamy Prabhawathi, Ponnurengam Malliappan Sivakumar, Mukesh Doble

**Affiliations:** Department of Biotechnology, Bhupat & Jyoti Mehta School of BioSciences, IIT Madras, Chennai, 600036, India; Agricultural University of Athens, GREECE

## Abstract

Contamination of food products by spoilage and pathogenic microorganisms during post process handling is one of the major causes for food spoilage and food borne illnesses. The present green sustainable approach describes the covalent immobilization of papain to LDPE (low density polyethylene), HDPE (high density polyethylene), LLDPE (linear low density polyethylene) and PCL (polycaprolactam) with curcumin as the photocrosslinker. About 50% of curcumin and 82-92% of papain were successfully immobilized on these polymers. After 30 days, the free enzyme retained 87% of its original activity, while the immobilized enzyme retained more than 90% of its activity on these polymers. Papain crosslinked to LLDPE exhibited the best antibiofilm properties against *Acinetobacter* sp. KC119137.1 and *Staphylococcus aureus* NCIM 5021 when compared to the other three polymers, because of the highest amount of enzyme immobilized on this surface. Papain acts by damaging the cell membrane. The enzyme is able to reduce the amount of carbohydrate and protein contents in the biofilms formed by these organisms. Meat wrapped with the modified LDPE and stored at 4°C showed 9 log reduction of these organisms at the end of the seventh day when compared to samples wrapped with the bare polymer. This method of crosslinking can be used on polymers with or without functional groups and can be adopted to bind any type of antimicrobial agent.

## Introduction

Foods are spoiled by the spoilage microflora, whereas the occurrence of outbreaks of foodborne diseases due to pathogen contaminated food is a global phenomenon. Therefore antimicrobial food packaging technologies have become more intense. Microbial contamination of food occurs mainly at their surface due to post process handling [1]. Antimicrobial active packages are those which are in contact with the food aiding in extending its shelf life by preventing the microbial growth [2]. Bacteriocins [3], organic acids [4], potassium sorbate [5] or pimaricin [6] exhibit antimicrobial activity within the food packaging materials. Immobilization of antimicrobials on food package rather than coating it on the surface of the wrapper reduces their amount required to achieve the antimicrobial effect as well as prolongs their activity.

Titanium dioxide, iron oxide, silver, gold and silver dioxide are examples of nanoparticle-based antimicrobials used in food wrap applications [7]. These are toxic [8] and affect the tissues in the human body. So, the use of natural antimicrobial agents including enzymes is in great demand. Peptides are emerging as new group of antibiotics and the antimicrobial nature of the one isolated from ovalbumin hydrolysate is reported [9]. Natural compounds including essential oils [10] and other herbal extracts have been tested as antimicrobial agents [11]. Peroxides, eugenol, nisin, lactoferrin, sodium diacetate, sorbic acid [12, 13], potassium sorbate, lysozyme [14], glucose oxidase [15] thymol, carvacrol [16], linalool and methylchavicol [17] are representative antimicrobial agents that have been found to inhibit the growth of food borne pathogens. Lysozyme loses its activity after immobilization on polyamide and ionomer films [15] which limits its use in food packs. Peroxides are toxic to humans and potassium sorbate shows toxicity towards animals. Essential oils and nisin exhibit poor antimicrobial activity against Gram negative bacteria. So there is a need for identifying novel compounds from natural sources which exhibit high activity and stability over a long duration of time. In addition there is a need to identify a non toxic cross linker to immobilize these compounds on the food wrap.

*Acinetobacter* spp. are aerobic and encapsulated Gram-negative bacilli which are contaminants found in a wide variety of products including pasteurized milk, frozen foods and chilled poultry [18]. *Acinetobacter* spp. biofilms play an important role in infectious diseases including periodontitis, bloodstream infections, and urinary tract infections. They are resistant to most of the commonly used antimicrobials and are recognized as one of the most difficult health associated infections to control and treat [19]. There are very few studies on preventing their contamination in frozen foods [20]. *Staphylococcus aureus* is a very common food borne pathogen which causes illness by producing heat stable enterotoxins [21].

Papain is an endolytic plant cysteine protease enzyme with high stability and activity under varying environmental conditions [22]. It exhibits proteolytic activity towards proteins, short-chain peptides, amino acid esters and amide links. Papain has an active site consisting of three residues namely, Cysteine-25, Histidine-159 and Asparagine-175 [23]. Initially, the substrate containing a peptide bond binds to the active site. The cys-25 gets deprotonated by His-159 and attacks the carbonyl carbon of the peptide chain. His-159 acts as a general acid, protonating the nitrogen in the peptide bond, which serves as the leaving group. After two more steps the carbonyl reforms to regenerate the enzyme. Asparagine-175 helps to orient the imidazole ring of His-159 to allow the deprotonation of Cys-25. Papain exhibits antifungal, antibacterial, anti-inflammatory and antibiofilm activities due to its proteolytic and elastolytic properties [24]. Hence it is used in several applications including debris removal in wound, chemo mechanical dental caries removal, to overcome allergies associated with leaky gut syndrome, hypochlorhydria (insufficient stomach acid) and gluten accumulation in the intestine as a result of insufficient pancreatic enzyme and stomach acid secretion [23]. Papain acts only in infected tissues in the tooth and breaks the partially degraded collagen present there, thereby removing dental caries [25]. Papain is mainly used in milk industry especially during cheese ripening for flavor development and milk coagulation [26], as a digestive and as an animal feed supplement, Literature reports on papain tested against food contaminant as food packages are minimal.

In the food industry, antimicrobial substances are used in the form of sprays or dips. But, such direct application has limited benefits because, the active substance is neutralized on contact with the food or it may diffuse rapidly from the surface into the food [27]. Whereas, immobilization of such substances to the surface of a polymer helps anchoring them to the material thereby preventing their movement into the food and hence, sustaining their activity and stability over a long period of time [28]. Covalent immobilization of an enzyme prevents its aggregation, proteolysis and interaction with the hydrophobic surface [28]. Currently, there is a strong interest in the use of renewable and nontoxic supports for immobilization to make the process more ecofriendly [29].

In this study, the immobilization of papain to LDPE (low density polyethylene), HDPE (high density polyethylene), LLDPE (linear low density polyethylene) and PCL (polycaprolactam) using curcumin as the cross linker is reported [30]. The current study is a green sustainable solution. LDPE is the most commonly used polymer in commercial films, carrier bags, protective foams and some flexible lids and bottles. It is a widespread material used for packing food on a daily basis [31]. Practically very little research is carried out on modifying surface of polyethylene food wrappers to impart antibacterial properties. One study describes the use of a bacteriocin produced by *Enterococcus casseliflavus* IM 416K1 entrapped in an organic—inorganic hybrid coating and applied to a LDPE film and tested as a food wrapper [32]. LDPE and LLDPE are flexible, while HDPE is rigid. All the three are used in food boxes. LDPE is used for making cling films and milk carton lining while LLDPE is used for stretch film. LDPE is more transparent than LLDPE and it is ideal for wrapping products which require visual observation. According to the 2011 data the global annual production of LDPE and LLDPE are 23.3 and 7.4 million tonnes indicating the easy availability of the former [33]. So experiments with food were performed with only one polymer, namely LDPE.

Photocrosslinking is a type of immobilization which involves the use of UV or visible light and has been extensively applied in metallic surface coatings, biomedical applications, drug delivery and tissue engineering [34]. High cost, as well as the toxic nature of currently used crosslinkers restricts their use in food packages since they come in contact with the food. Commonly used photochemical reactive groups include aryl azides and diazirines which are explosophores and toxic [35]. Curcumin is tested as a crosslinking agent in the current study, since it is safe and widely used as a food flavoring agent. This is the first report in which it is studied for its potential as a photocrosslinker. It has several functional groups and is a well known antioxidant and anti-inflammatory agent and has medicinal benefits against several diseases including cancer and diabetis [36]. Even though crosslinking of polymers with high energy radiation has been tested for many other applications, use of photocrosslinking technology for food pack applications is minimal. In the present study, when curcumin and polymer are UV treated, they form biradicals. Papain crosslinks to these biradicals in the presence of UV light to form papain immobilized polymer with curcumin as the linker.

## Materials and Methods

### Bacterial strains and chemical materials

A bacterium was isolated from cottage cheese using serial dilution procedure, by repeated streaking to obtain isolated colonies. It was later identified through 16S rDNA analysis (Genie (India) Ltd, Banglore) as *Acinetobacter* sp. KC119137.1. (Fig A in S1 File). An isolated colony was inoculated in 25ml of nutrient broth (Himedia, item no: M002) and incubated at 37°C for 16 h in a shaker. 0.6ml of the above culture was added to 0.4ml of 60% sterile glycerol (SRL, item no: 072929) and aliquots of these were maintained as glycerol stocks at -20°C and sub-cultured whenever needed. Same procedure is followed for the other strain used in this study, namely, *Staphylococcus aureus* NCIM (National Collection of Industrial Microorganisms) 5021. It was purchased from the National Chemical Laboratory (NCL), Pune, India. Papain (Super Religare Laboratories (SRL), item no: 164739). Curcumin (Sigma-Aldrich, item no: C1386), and all other chemicals and reagents used in this study were purchased from Sigma (St. Louis, MO), Super Religare Laboratories (SRL), and HiMedia (Mumbai, India). Polycaprolactam was purchased from marine industrial polymers, Chennai, India., HDPE, LDPE and LLDPE sheets (0.175 micron thickness) were purchased from Industrial Insulations Ltd, Chennai, India.

### Papain and curcumin estimation

The activity of papain was determined by using a reported procedure [37] using casein (SRL, item no: 034023) as the substrate. Curcumin was dissolved in ethanol (China Changshu Yang yuan Chemical, batch no: 20140720) and its concentration was estimated by using a reported method [38] with the help of an UV spectrophotometer (Perkin-Elmer, Lambda 35, Shelton, CT).

### Determination of Minimum inhibitory concentration (MIC)

The MIC values of curcumin, papain and a mixture of curcumin and papain (1:2.5 by wt) against both the bacterial strains were determined by the microdilution broth assay method [39] with slight modifications as reported by Sarker et al [40] using resazurin (Sigma-Aldrich, item no: R7017) as an indicator. The colour change was assessed visually and the highest dilution that remained blue (inhibition of growth) indicated the minimum inhibitory concentration of the compound. A colour change from blue to pink showed the growth of the organism.

### UV crosslinking and characterization

The photochemical cross-linking of papain to LDPE, HDPE, LLDPE and PCL surfaces (1x1cm) was performed in two stages at 30°C in a rectangular cabinet (Superfit, India) in the presence of air, by exposing them to UV light at 365 nm and 500 W. The distance between the UV source and the film was 20 cm. Curcumin was dissolved in ethanol and 200 μl of this solution containing 5.43 μM of it was spread on these polymer films using a spin coater (Apex instruments co pvt ltd, India), followed by UV treatment for 24 hours to form CC (curcumin cross linked)-LDPE, CC-HDPE, CC-LLDPE and CC-PCL. In order to calculate the amount of curcumin crosslinked to these polymers, the curcumin crosslinked polymers were taken separately in four different tubes and washed with 25mM of phosphate buffer solution at a pH of 7 and the curcumin left in the washing solution was quantified. This was then subtracted from the curcumin initially taken for crosslinking. 0.1 mM of papain solution at a pH of 7 was then spin coated onto these surfaces and UV treated for 10 minutes to form PCC (Papain immobilized curcumin crosslinked)-LDPE, PCC-HDPE, PCC-LLDPE and PCC-PCL. The efficiency of the cross linking process and the activity of the enzyme retained after immobilization were estimated from the following formulae.

$$Immobilzation\;efficiency\;=\;\left(1-\frac{P_{s}}{P_{o}}\right)x\;100$$

Po is the initial concentration of papain prepared to coat the polymers; Ps is the papain concentration in the washing solution left after washing the PCC-polymers.

$$Activity\;recovery\;\left(or\;retained\right)after\;immobilization\;=\;(Activity\;of\;immobilized\;enzyme/Initial\;activity\;of\;free\;enzyme)\;x100$$

The polymers were stored at 4°C for 30 days to study their stability i.e. the activity retained by the enzyme after storage for 30days. The papain stability was calculated as,

papain stability = (enzyme activity after storage for 30 days/Initial enzyme activity) x 100

The polymers were washed in PBS buffer (pH of 7.0), and then the enzyme activity was estimated. This was repeated for seven cycles and the enzyme activity was calculated at the end of this washing process as follows.

Recycling efficiency = (Enzyme activity in the 7th cycle/activity in the first cycle)100

### Physicochemical characterization of the films

The changes in the structure of the polymers, and the effect of photo crosslinking and the immobilization of the enzyme were identified from the Fourier Transform Infrared (FTIR) spectra recorded in the frequency range of 500–4000 cm^-1^ using a Perkin-Elmer PE 1600 FTIR spectrometer. The elemental composition of the polymers’ surfaces after the immobilization were determined using a scanning electron microscope (SEM) equipped with a energy dispersive x-ray spectroscope (EDAX) (JEOL JSM 5600 LSV model, supplied by JEOL, Tokyo, Japan).

Contact angle of these polymers were measured using a Goniometer (Kruss germany) with Milli-Q water (Millipore grade). The images obtained were analyzed with a Digital Scrapbook Artist 2 Software (DSA2) to determine the static and dynamic contact angles (SW4001), with an accuracy of ±0.1°.

### Biofilm formation and characterization

Each bacterial strain was inoculated from the stock culture into 25 ml of nutrient broth and incubated at 37°C for 16 h in a shaker (Scigeneis Pvt., Ltd, Chennai, India) at 120 rpm. A total of 500 μl from the above preculture was inoculated into 50 ml of nutrient broth and cultured under the above conditions. After 16h the culture broth was taken in sterile falcon tube and was centrifuged (Eppendroff, Germany) at 4°C at 4480 rcf (relative centrifugal force) for 10 min. The pellet was diluted in phosphate buffer solution (10 mM) and its optical density (OD) value was adjusted to 0.1 (at 600 nm) which was equivalent to approximately 1 x 10^7^ cells/ml. Each bacterial suspension was subsequently inoculated into three flasks containing nutrient broth along with bare, CC and PCC polymers (of size 1x1cm). These flasks were stirred for 24 h at 30°C under shaking at 120 rpm using an Orbitek shaker (Scigeneics India ltd, India). Following this incubation period, the samples were removed with sterile forceps and were washed twice with sterile water to remove the unbound cells. The samples were subsequently inoculated in sterile tubes containing 0.7% of saline solution. The biofilm formed on the surface of each polymer was carefully dislodged by water-bath ultra sonication (Thosan Pvt., Ltd, Ajmer, India) for a total of 10min with 1min interval [41].

The protein content in each of the biofilms was estimated by the Lowry’s method [42] using crystalline bovine serum albumin as the reference standard. The exopolysaccharides content present in each of the biofilms was estimated by the phenol sulfuric acid method using glucose (SRL, item no: QK1Q610671) as the standard [43]. The live bacterial colonies in the biofilm was removed and their number was determined as per a standard procedure and represented as colony forming units (CFU/cm^2^ of the polymer surface) [43].

### Morphology of the biofilms

The bare, CC-LDPE, PCC-LDPE polymer surfaces were washed with distilled water and the biofilms were fixed using 3% of glutaraldehyde (in 0.1% phosphate buffer at a pH of 7.2) for 1 h [43]. They were then rinsed twice with phosphate buffer, once with distilled water, dried overnight in a desiccator, coated with gold at 30 mA for a minute, and viewed under a scanning electron microscope SEM (JEOL JSM 5600 LSV model, supplied by JEOL, Tokyo, Japan).

The live and dead microbial cells present on the polymers surface were determined using a mixture of two nucleic acid fluorescent staining dyes containing SYTO9 and propidium iodide (PI) (LIVE/DEAD *Bac*Light Bacterial Viability Kit, Invitrogen, USA). The former dye stains both live and dead cells as green while the latter dye penetrates the wall of the damaged cells and binds to DNA and appears as red. The polymer films (bare, CC-LDPE and PCC-LDPE) were individually inoculated into flasks containing 25 ml of nutrient broth. Then 1ml of each bacterial suspension (approximately 10^7^ cells) was subsequently inoculated into these flasks. Flasks were stirred for 24 h at 30°C under shaking at 120 rpm. The samples were removed with sterile forceps and were washed twice with sterile water to remove the unbound cells. These films were stained with the dye mixture and then observed under a fluorescence microscope (Leica DM5000, Germany) [43].

### BATH Assay

BATH assay was performed on both bacteria to determine the hydrophobicity of each bacterial surface using a standard procedure [44].

### Food packaging experiment

A slight modification to the methodology reported by Besse et al [45] was followed here. Freshly processed beef sample was purchased from a supermarket and kept frozen at -20°C and thawed at 2°C for 1 day before use. It was then cut into small squares, each weighing 1 g, and was inoculated with 10^7^ cells of *Acinetobacter* sp. and *S*.*aureus*, separately. Samples were left undisturbed for 5 min for the inoculum to soak in and the cells to attach. They were subsequently wrapped in LDPE, CC-LDPE and PCC-LDPE, then placed in a petri plate and incubated at 4°C. After 7 days, the meat samples were opened aseptically and approximately 0.2g were homogenized in 1ml of 0.7% saline solution and the numbers of viable bacteria present on the meat samples were estimated as described below. 100μl of this solution was serially decimally diluted and subsequently spread-plated on nutrient agar (Himedia, item no: M001) plates using a L shaped glass rod. After 24h of incubation at 37°C, the viable colonies were counted visually on nutrient agar plates and represented as colony forming units (CFU/g of beef).

### Statistics

All the analysis was repeated thrice on three independent samples and the data was reported as means ± standard errors. One way ANOVA and two sample t-test were performed using MiniTab Ver 14.0 (MiniTab inc, USA). A p value <0.05 was considered to be statistically significant.

## Results and Discussion

### Physicochemical characterization of the films

**Fig 1:**
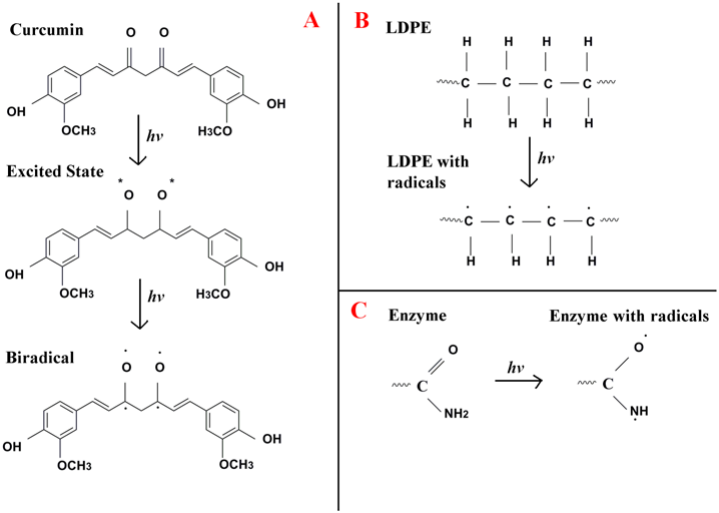
The chemical changes which occurred on UV treatment of (A) curcumin, (B) LDPE and (C) enzyme.

**Fig 2:**
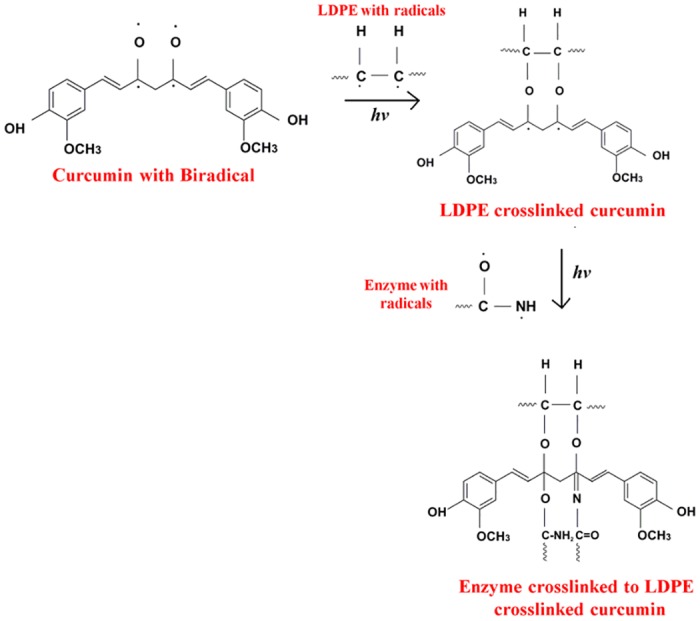
Step by step reactions which lead to the formation of Papain crosslinked LDPE.

As represented in Fig 1, UV treatment leads to the formation of radicals in curcumin, LDPE and papain [43] with subsequent changes in their chemical structures. The formation of papain immobilized LDPE with curcumin as a crosslinker involves two steps. When curcumin coated LDPE is exposed to UV light, the biradical of curcumin reacts with the carbon radical of LDPE in the presence of UV to form CC-LDPE, through C-O-C bond (Fig 2). When this curcumin crosslinked LDPE is coated with UV treated papain and exposed to UV light, the former reacts with O and N radicals of the papain leading to the formation of CO and CN covalent crosslinkages. This ultimately leads to the formation of papain crosslinked LDPE with curcumin as a crosslinker (PCC- LDPE) (Fig 2) [46]. This reaction was confirmed by FTIR spectra.

**Fig 3:**
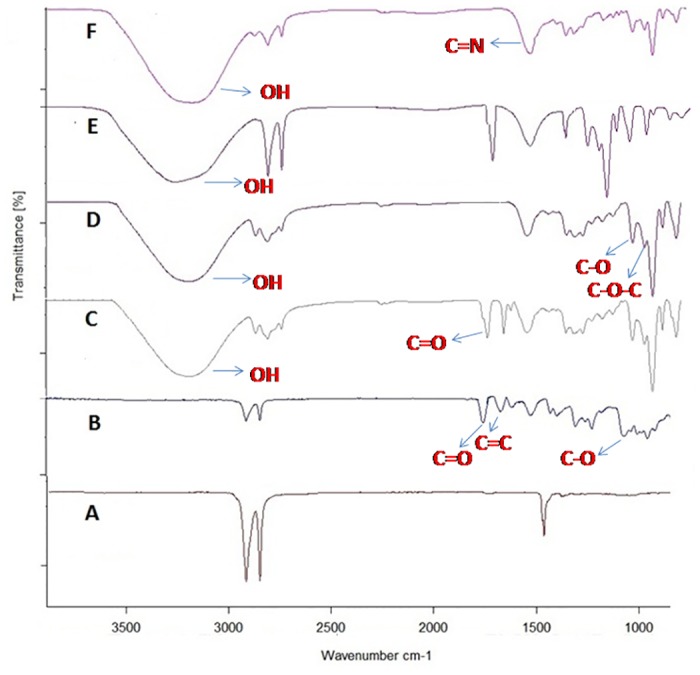
FTIR spectra of A) Non UV treated LDPE, B) UV treated LDPE, C) Non UV treated CC-LDPE, D) UV treated CC-LDPE, E) Non UV treated PCC-LDPE and F) UV treated PCC-LDPE.

The FTIR spectra of the LDPE (Fig 3A) has bands at 1462cm^-1^ which corresponds to methylene and methyl groups respectively. After UV treatment, new bands are formed at 1734 and 1640 cm^-1^ (Fig 3B) indicating the formation of C = O and C = C groups respectively [47]. FTIR spectrum of curcumin coated LDPE (before UV treatment) (Fig 3C) shows bands corresponding to OH (3290 cm^-1^), C = C (1626 cm^-1^) and C = O (1743 cm^-1^) groups, which are present in curcumin. These results are in agreement with earlier reports [48,49]. Presence of enol peaks at 1078 cm^-1^ and 1136 cm^-1^ (after UV treatment) indicates that curcumin has crosslinked to the polymer through an oxygen group. The band corresponding to OH group in the non uv treated polymer (3290 cm^-1^) shifts to 3167 cm^-1^ in the UV treated sample. Bands at 1335 cm^-1^ and 1379 cm^-1^ indicate the CH_3_ bending vibration present in non UV treated polymer which disappears when curcumin is crosslinked to LDPE [49]. Peak at 1050 cm^-1^, represents C-O-C group which has appeared after the crosslinking of curcumin to LDPE. Appearance of 1272 cm^-1^ peak indicates C-C group. The carbonyl group of curcumin (C = O) observed at 1743 cm^-1^ in non UV treated sample gets converted to C-O (1150 cm^-1^) in the UV treated sample. The appearance of the peak at 1150 cm^−1^ (after UV treatment) can be attributed to the crosslinking of curcumin to LDPE (Fig 3D)

The FTIR spectra of papain coated on CC-LDPE before (Fig 3E) and after UV treatment (Fig 3F) indicates the formation of C = N group (1624 cm^-1^) in the latter which confirms the covalent immobilization of papain to CC-LDPE [38]. Appearance of peak at 1042 cm^-1^ represents C-C which is less intense in non UV treated than in the UV treated sample indicating the formation of several carbon-carbon bonds between papain and CC-LDPE. This also indicates the crosslinking of COOH group in papain to CC-LDPE. The band at 1529 cm^-1^ indicates the presence of amide group in PCC-LDPE [50]. Bands at 1042, 1136 and 1285 cm^-1^ indicate the presence of primary amine (CN stretch) and peak at 1624 cm^-1^ indicates the formation of secondary amine (NH) which arises due to the immobilization of papain to LDPE [49]. These changes in the FTIR spectra confirm that the papain is covalently crosslinked to CC-LDPE. The photochemical crosslinking when compared to chemical process is operated at room temperature and is easy to control, which is helpful in preserving the 3-dimensional structure of the enzyme after UV treatment [51].

Most of the commonly used photocorsslinkers include aryl azides and diazirines as their reactive groups. These are toxic and hazardous [35]. So, their application in food and medical industry is limited, which necessitates the need for nontoxic photocrosslinker. In this study, curcumin is tried as a novel photocrosslinker. Polyethylene is a polymer widely used for many applications including food packages. Lack of functional group limits it from being used as a base for immobilizing antimicrobials and proteins on its surface. In the present study, UV treatment of curcumin as well as LDPE results in the formation of biradicals, favoring the crosslinking between them and then later to papain. The successful crosslinking of curcumin and further immobilization of papain to LLDPE, HDPE and PCL are confirmed similarly from their respective FTIR spectra (Figs B to D and Table A in S1 File). This is the first report on the use of curcumin as a photocrosslinker. Curcumin could be used to crosslink surfaces that could be used for various applications including food, pharmaceuticals and medicine which would require the use of non-toxic crosslinker.

### Elemental composition of the surfaces concentration and morphology of the polymers

The changes in the elemental composition of the polymers’ surfaces after UV crosslinking were investigated by EDAX. These measurements indicated 98.1±0.49 and 1.5±0.18 weight % of elemental carbon and oxygen, respectively, on the surface of UV treated LDPE and 92.5±1.80 and 7.5±0.69 weight % of elemental carbon and oxygen, respectively, on the surface of CC-LDPE respectively. The increase in percentage of oxygen in the latter is due to the immobilization of curcumin which possesses several oxygen groups. 67.0±3.70, 13.3±1.60, 18.7±2.20 and 0.1±0.06 weight % of elemental carbon, nitrogen, oxygen and sulphur respectively are present on the surface of PCC-LDPE. The appearance of elemental sulphur and nitrogen are due to the immobilization of the enzyme which contains amino acids, once again emphasizing its presence on the polymer surface.

The contact angle of LDPE, CC-LDPE and PCC-LDPE were 128± 2.3°, 80± 1.8° and 71±1.3° respectively. The relevant results for HDPE, LLDPE and PCL were 110± 2.7°, 83± 2.6° and 65±1.9°; 100± 3.2°, 85± 2.3° and 70±2.5° and 79± 1.9°, 70± 1.4° and 58±1.3° respectively. It is observed that PCL is the most hydrophilic and LDPE is the most hydrophobic surface. Non treated polymers are the most hydrophobic while the crosslinking successively reduces the hydrophobicity. Previously, it has been reported that the hydrophilic surfaces generally reduce the adhesion of microorganisms [52].

### Stability and activity of immobilized enzyme

The percentages of curcumin and papain immobilized on the four polymers are listed in Table 1. It can be concluded (based on the one way ANOVA, p<0.05) that the percentage of curcumin and papain crosslinked to LLDPE is the highest, followed by their amount on PCL, LDPE and HDPE. After 30 days of storage, free enzyme retained 87.5 ±2.0% of activity, while PCC-LDPE retained 93.0±1.8% of enzyme activity, PCC-HDPE, PCC-LLDPE and PCC-PCL retained 89.2±1.6, 97.3±2.2 and 95.0±2.1% of enzyme activity respectively (Table 1). These data show that both the enzymatic activity and stability are well maintained after crosslinking.

**Table 1:** The weight percentages of curcumin and papain immobilized on the four polymers.

POLYMER	CURCUMIN %	ENZYME %	Activity retained after 30 days
LDPE	50±2.6	86.3±1.5	93.0±1.8
HDPE	48±3.5	82.3±3.4	89.2±1.6
LLDPE	60±1.5	92.4±2.2	97.3±2.2
PCL	59±4.1	90.0±1.2	95.0±2.1

### Biofilm inhibition

The MIC values of papain, curcumin and the mixture of papain and curcumin needed to inhibit the growth of *Acinetobacter* sp. as determined by microdilution broth assay method [53] were 7.80±0.18, 15.60±0.28 and 0.98±0.11 μM respectively. For *S*.*aureus*, the corresponding MIC values of papain, curcumin and a mixture of papain and curcumin were 1.95±0.22, 3.90±0.37 and 0.98±0.11 μM respectively. The combination of papain and curcumin exhibited enhanced activity than the individual compounds when used alone.

**Fig 4:**
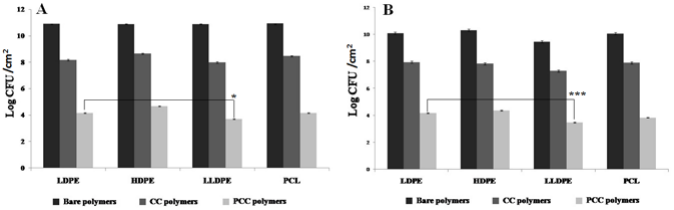
Population (Log CFU/cm^2^) of (A) *Acinetobacter* sp. and (B) *S*.*aureus* biofilms formed on bare, CC and PCC polymers after 24 hours of incubation (*p<0.5,***p<0.001).

Papain immobilized curcumin crosslinked polymers (PCC) showed the least number of *Acinetobater* sp. and *S*.*aureus* attached cells (Fig 4A & 4B). Whereas, maximum number of attached cells were observed on the bare polymers. It is observed that PCC-LLDPE showed the best antimicrobial activity against both species (maximum reduction in the number of live biofilm cells) followed by PCL, LDPE and then HDPE (p<0.5 for *Acinetobater* sp. and p<0.001 for *S*.*aureus*). These results negatively correlate with the percentage of enzyme immobilized on the polymers (correlation coefficient between live colony count on the polymer surface and percentage of enzyme immobilized on the polymer surface = -0.85 for *Acinetobater* sp. and -0.89 for *S*.*aureus*). Highest percentage of enzyme was immobilized on LLDPE and the lowest on HDPE (Table 1). Percentage of enzyme crosslinked to LLDPE was comparatively more, which could probably be due to the presence of more short branches (more atoms/mol) in this polymer when compared to that in LDPE and HDPE. Since HDPE contains no branches, it shows less crosslinking when compared to LLDPE and LDPE. Presence of C = O as well as NH groups in PCL, permits more radical formation leading to increased curcumin and enzyme crosslinking when compared to LDPE and HDPE. These results demonstrate that photo-cross-linking is robust and can dramatically improve the structural stability of the enzyme. The conversion of weak ionic bonds to strong covalent bonds prevents the leakage of the enzyme leading to its high activity and stability [54].

*Acinetobacter* is a contaminant found in many food products and it is a challenge to eradicate it from contaminated food. Papain crosslinked polymer reduces its growth. This enzyme is also effective against *S*.*aureus* which is a common food borne pathogen. Veluchamy et al. have reported superior performance of subtilisin (a bacterial protease) immobilized on polycaprolactum with glutaraldehyde against *Escherichia coli* and *S*.*aureus* [41,55]. Orgaz et al. reported the effect of pronase treated chitosan against several foodborne pathogens. They showed 8.0, 7.5, 6.0, 5.0 and 0.5 log reduction against *Bacillus cereus*, *L*. *monocytogenes*, *P*. *fluorescens*, *S*.*enterica and S*.*aureus* respectively [55]. In another study, Morvay et al. reported that protease from *Bacillus licheniformis* inhibited the formation of mature biofilms of *B*.*cereus* and *Pseudomonas aeruginosa* [56]. Lysozyme was covalently attached to polystyrene resin beads by the sole histidine residue (His-15) through peptide spacers of various lengths. Three 6-aminocaproic acid units of spacer length displayed the greatest degree of hydrolytic activity against *Micrococcus lysodeikticus* [57]. Prabhawathi et al. reported 2 and 7 times reduction in carbohydrate and 9 and 5 times reduction in biofilm protein of *S*.*aureus* and *E*. *coli* respectively on lipase immobilized polycaprolactam (LIP) when compared to uncoated polycaprolactam (UP) [58]. The immobilization was performed using Langmuir Blodgett technique. An arginine—tryptophan-rich peptide (CWR11) immobilized on a silicone surface displayed antimicrobial activity against a broad spectrum of microbes such as *S*.*aureus*, *E*.*coli* and *P*.*aeruginosa* via wall disruption [59].

**Fig 5:**
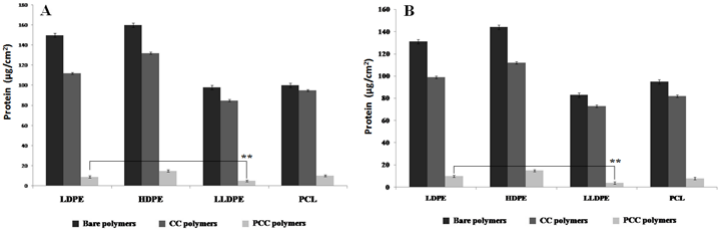
Protein content of (A) *Acinetobacter* sp. and (B) *S*.*aureus* biofilms formed on bare, CC and PCC polymers after 24 hours of incubation (**p<0.01).

Bacteria enclosed in biofilms are usually more resistant to antimicrobial treatments, when compared to the same bacteria in planktonic form [60]. Since the major components of the biofilm matrices are exopolysaccharides and proteins, the action of the enzyme in reducing their amount is investigated here. Biofilms were developed by the adhesion of microbial cells on the polymer surface. The subsequent colonization of the organisms then is facilitated through the production of exopolysaccharides. Exopolysaccharides generally account for 50–90% of the total organic carbon in the matrix [41]. All the four PCC surfaces had less biofilm protein followed by the four CC surfaces (Fig 5). The bare polymer surfaces had the highest protein content. The amount of protein in the biofilm formed on PCC-LLDPE was lesser than that on LDPE (p < 0.01). Papain increased the membrane permeability as well as acted on the proteins and the peptidoglycan present on the outer membrane of the bacteria [23], leading to the loss of cell contents, and thus, resulting in bacterial destruction. There are literature reports which show that protease [41,61] hydrolyses the protein present on a polymer surface.

**Fig 6:**
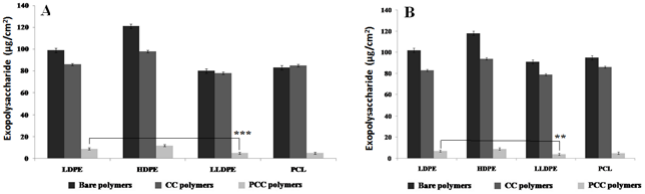
Exopolysaccharide content of (A) *Acinetobacter* sp. and (B) *S*.*aureus* biofilms formed on bare, CC and PCC polymers after 24 hours of incubation (**p<0.01, ***p<0.001).

All the four types of polymers when crosslinked with papain showed reduced exopolysaccharide content in their biofilm and once again this content in the biofilm formed on PCC-LLDPE was lesser than that on LDPE (p<0.01 for *Acinetobater* sp. and p<0.01 for *S*.*aureus*) (Fig 6). Reduction in exopolysaccharide content could disturb the uniformity and integrity of the biofilm structure which could lead to its breakdown as observed in the present study. A strong correlation is observed between the reduction of CFU with reduction in protein content in the biofilm (correlation coefficient > 0.95) as well as reduction of CFU with reduction in exopolysaccharide content in the biofilm (correlation coefficient > 0.91) for both the organisms with all the four polymers. Maximum reduction of exopolysaccharide and protein were observed on LLDPE surface and least on HDPE surface.

**Fig 7:**
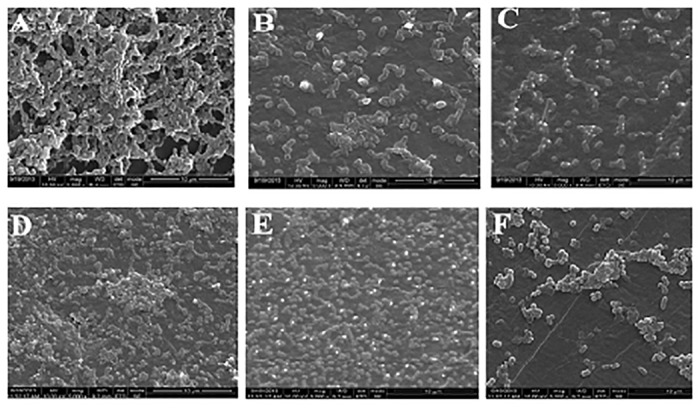
SEM images of *Acinetobacter* sp. grown on (A) LDPE (B) Curcumin crosslinked LDPE (C) Papain immobilized CC-LDPE and *S*.*aureus* grown on (D) LDPE (E) Curcumin crosslinked LDPE (F) Papain immobilized CC-LDPE.

**Fig 8:**
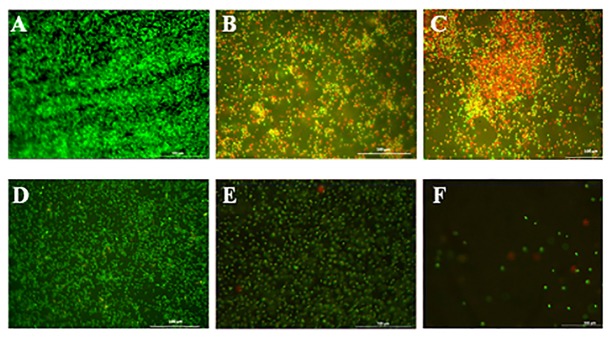
Fluorescence microscopic images of *Acinetobacter* sp. biofilm on (A) LDPE (B) curcumin crosslinked LDPE (C) papain immobilized CC-LDPE (Green-live cells, red- dead cells due to cell membrane damage) and *S*.*aureus* biofilm on (D) LDPE (E) curcumin crosslinked LDPE (F) papain immobilized CC- LDPE.

SEM images of the biofilm of *Acinetobacter* sp. (Fig 7A, 7B & 7C) and *S*.*aureus* on LDPE, CC-LDPE and PCC-LDPE (Fig 7D, 7E & 7F) support the CFU results. Least number of bacteria was observed on PCC-LDPE and maximum on bare LDPE. Cells with compromised and damaged membrane that are considered to be dead are stained red by the Backlight dye, whereas the cells with intact membrane are stained green. Fluorescence images show more live *Acinetobacter* sp. and *S*.*aureus* cells on LDPE surface (Fig 8A & 8D), a mixture of live and dead cells on CC-LDPE (Fig 8B & 8E) and more dead cells on PCC-LDPE (Fig 8C & 8F). These data indicate that the enzyme is able to damage the cell membrane and hence reduce the number of live bacterial cells in the biofilm [41].

Also, contact angle indicates that bare polymer is more hydrophobic than the enzyme immobilized one and so less bacterial attachment is expected on the latter. It could be observed from CFU data, and SEM and fluorescence microscopic analysis that there is more adhesion of microbes to bare polymer surface than enzyme immobilized surface. So less attachment of bacteria and biofilm are observed on the enzyme immobilized surface because of the antibacterial activity of papain as well as the relatively hydrophillic nature of this surface

### Characterization of bacterial surface properties

Bacterial surface properties, such as the hydrophobicity, are considered important factors influencing the adherence of cells to biomaterial surfaces and this property is determined from the BATH assay. As the concentration of hexadecane is increased, the O.D value corresponding to *Acinetobacter* sp. decreased indicating that more of this organism is partitioning to the hydrophobic solvent phase while the reverse trend to be observed with *S*. *aureus*. (Fig F in S1 File). This indicates that the former organism is relatively more hydrophobic than the latter. Hydrophobic bacteria tend to adhere more to hydrophobic biomaterial surfaces. More number of *Acinetobacter* sp. *is* attached to the bare surface than *S*.*aureus*, probably because the former is more hydrophobic than the latter. The cell membrane of *Acinetobacter* spp. consists of proteins, lipopolysaccharides and phospholipids. Its hydrophobicity is due to the presence of pilli, lipopolysaccharides and hydrophobic amino acids in the flagella. The protease enzyme has probably acted on the cell membrane of *Acinetobacter* sp. thereby altering its surface properties leading to its reduced adhesion on the polymer.

### Food packaging experiment

**Fig 9:**
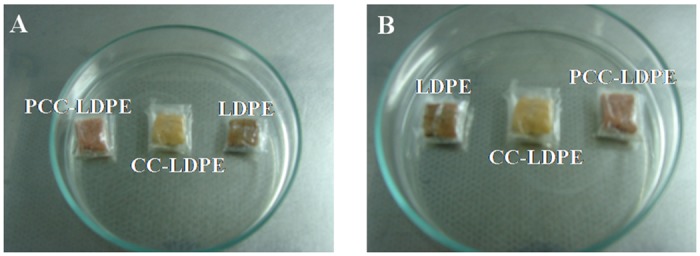
Effect of wrappers formed of LDPE, CC-LDPE and PCC-LDPE on meat samples, inoculated with A) *Acinetobacter* sp. and B) *S*.*aureus* after 7 days of storage at 4°C.

Antimicrobial action of PCC-LDPE was tested on beef samples against both the bacterial strains. The number of live *Acinetobacter* cells in the meat samples wrapped with LDPE, CC-LDPE and PCC-LDPE at the end of 7^th^ day represented as log(CFU/g of beef) were 11.7±11.1, 7.8±7.2, and 2.5±2.1 respectively indicating the effectiveness of the enzyme (p < 0.001) in reducing the number of live bacteria in the food. The number of live *S*.*aureus* cells in the meat samples wrapped with the same polymers at the end of 7^th^ day represented as log(CFU/g of beef) were 11.1±10.6, 7.7±6.9 and 1.8±1.5 respectively, once again indicating the effectiveness of the enzyme (p<0.001). Of course, even simple crosslinking of curcumin to LDPE also leads to 4 log reduction in the number of live bacteria because of the antibacterial nature of the curcumin. Sung et al. reported a 5 log reduction in the growth of *L*.*monocytogenes* at the end of 6 days on beef loaves when it was wrapped with garlic oil incorporated LDPE films [62]. Hauser & Wunderlich reported the effect of sorbic acid coated polyethylene polyamide films on *E*.*coli* DSM 498 contaminated cheese [12]. This coating reduced the growth of this organism at the end of 4 weeks by a factor of ten. The same study when conducted on *E*.*coli* contaminated pork, resulted in 4 times reduction of the contaminant at the end of 7 days. Antimicrobials such as allyl isothiocyanate, garlic oil, rosemary oil and trans-cinnamaldehyde were incorporated in soy protein isolate and then were coated on oriented polyethylene/polypropylene (OPP/PE) packages for extended shelf life of alfalfa, broccoli and radish sprouts. The total microbial growth at the end of 5 days decreased significantly when compared to the uncoated sample [63]. 10% of lauramide arginine ethyl ester when coated on ethylene-vinyl alcohol copolymers film showed 4 log reduction in the growth of *L*.*monocytogenes and S*. *enterica* at the end of 6 days. The coating showed higher antimicrobial activity against Gram positive bacteria than against Gram negative bacteria [1]. Beef samples wrapped with plastic bags had 1000, 10,000 and 1,000,000 colonies of *Brochotrix thermosphacta*, *Enterobacteriaceae* and *Carnobacterium* spp. per gram of sample respectively at the end of 32 days of storage at 1°C. Whereas, samples wrapped with plastic bags coated with a mixture of nisin, EDTA and HCl showed 100, 100 and 10,000 colonies of the same organisms respectively [64,65]. All these studies indicate that Gram negative bacteria are highly resistant to antimicrobial agents when compared to Gram positive bacteria. The present study indicates that papain exhibits activity against both the Gram negative *Acinetobacter* sp. KC119137.1 as well as the Gram positive *S*. *aureus*. It is seen from the images (Fig 9A & 9B) that, the meat sample wrapped with enzyme immobilized LDPE (PCC-LDPE) appears to be fresh when compared to the one wrapped with bare polymer. Growth of bacteria could be seen on the latter which indicates the spoilage of the food.

## Conclusion

Curcumin has been successfully used as a novel photo crosslinker to covalently couple papain to four different types of polymers. These papain crosslinked polymers remained stable and active for a period of 30 days. Moreover, the treated surface turned hydrophilic, thereby becoming resistant to adhesion of microbes to it. It also exhibited antibiofilm activity against both a Gram positive, as well as a Gram negative strain. Literature reports generally indicate antibacterial coatings that act only on one type of organism but in the approach described here, the enzyme acts non-specifically on the cell membrane of both types of bacteria. Microorganisms generally develop resistance to antibacterial agents due to the alterations in their genes but the components of the cell membrane remain unaltered and hence the latter could be a good target as demonstrated here. Papain crosslinked polymers show excellent antibacterial activity against *Acinetobacter sps*. KC119137.1 and *S*.*aureus* once these have contaminated beef (more than 9 log reduction in the number of live bacteria), due to the combined effect of the crosslinked curcumin and the immobilized papain. This strategy provides a promising platform for fabricating robust, nontoxic, renewable and low cost antimicrobial films, which may offer a wide range of applications in food, pharmaceutical and biomedical packaging industries. This strategy can be applied for coating surfaces which do not have any functional groups on them. Both, curcumin and papain, reported here are food-based products and hence can be considered as safe. Further studies need to be done with other food-borne bacteria, as well as aiming to study the effect of this enzyme on food quality (including changes in texture and colour) before it can be taken up for commercial applications.

## Supporting Information

S1 File Fig. A, The phylogenetic tree of *Acinetobacter* sps. KC119137.1. (FM1). Fig. B, FTIR spectra of A) Non UV treated PCL, B) UV treated PCL, C) Non UV treated CC-PCL, D) UV treated CC-PCL, E) Non UV treated PCC-PCL and F) UV treated PCC-PCL. Fig. C, FTIR spectra of (A) Non UV treated HDPE, (B) UV treated HDPE, (C) Non UV treated CC-HDPE (D), UV treated CC-HDPE, (E) Non UV treated PCC-HDPE and (F) UV treated PCC-HDPE. Fig. D, FTIR spectra of (A) Non UV treated LLDPE, (B) UV treated LLDPE, (C) Non UV treated CC-LLDPE, (D) UV treated CC-LLDPE, (E) Non UV treated PCC-LLDPE and (F) UV treated PCC-LLDPE. Fig. E, Organism hydrophobicity with bath assay. Table A, Table for FTIR.
(DOC)
