# Supplementary material for: Design of a Papain Immobilized Antimicrobial Food Package with Curcumin as a Crosslinker
Source: PLoS One. 2015 Apr 23;10(4):e0121665. doi: 10.1371/journal.pone.0121665 (PMC4408049; doi:10.1371/journal.pone.0121665)
Supplement: S1 File — Fig. A, The phylogenetic tree of Acinetobacter sps. KC119137.1. (FM1). Fig. B, FTIR spectra of A) Non UV treated PCL, B) UV treated PCL, C) Non UV treated CC-PCL, D) UV treated CC-PCL, E) Non UV treated PCC-PCL and F) UV treated PCC-PCL. Fig. C, FTIR spectra of (A) Non UV treated HDPE, (B) UV treated HDPE, (C) Non UV treated CC-HDPE (D), UV treated CC-HDPE, (E) Non UV treated PCC-HDPE and (F) UV treated PCC-HDPE. Fig. D, FTIR spectra of (A) Non UV treated LLDPE, (B) UV treated LLDPE, (C) Non UV treated CC-LLDPE, (D) UV treated CC-LLDPE, (E) Non UV treated PCC-LLDPE and (F) UV treated PCC-LLDPE. Fig. E, Organism hydrophobicity with bath assay. Table A, Table for FTIR. (DOC) [file pone.0121665.s001.doc]

**Figure A: The phylogenetic tree of *Acinetobacter* sps. KC119137.1. (FM1)**

**
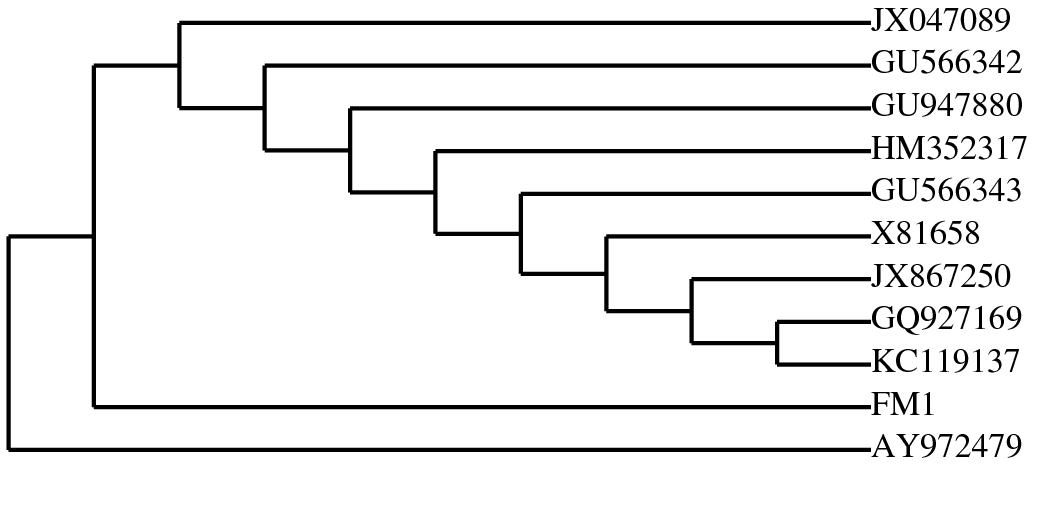
**

**Figure B:** FTIR spectra of A) Non UV treated PCL, B) UV treated PCL, C) Non UV treated CC-PCL, D) UV treated CC-PCL, E) Non UV treated PCC-PCL and F) UV treated PCC-PCL.

**
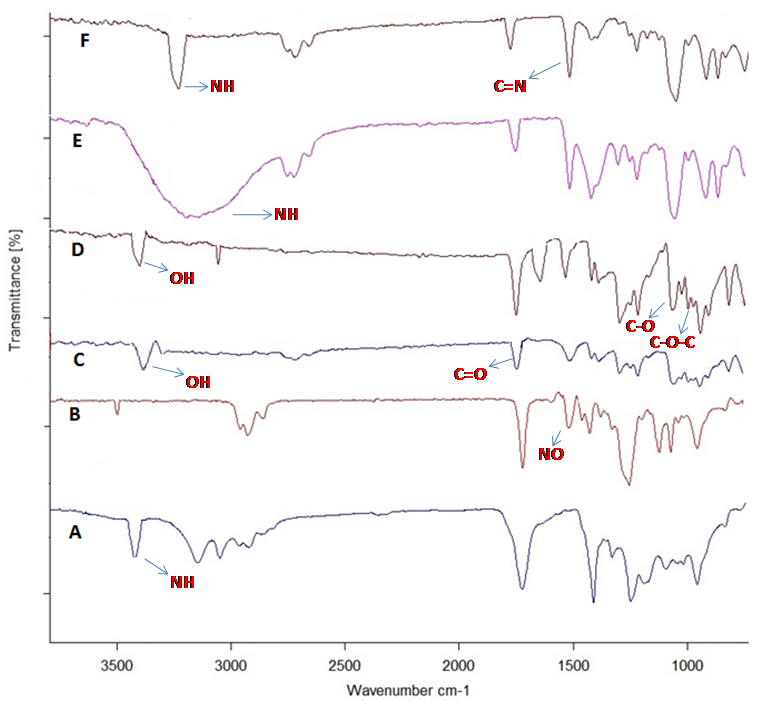
**

**Figure C:** FTIR spectra of (A) Non UV treated HDPE, (B) UV treated HDPE, (C) Non UV treated CC-HDPE (D), UV treated CC-HDPE, (E) Non UV treated PCC-HDPE and (F) UV treated PCC-HDPE.

**
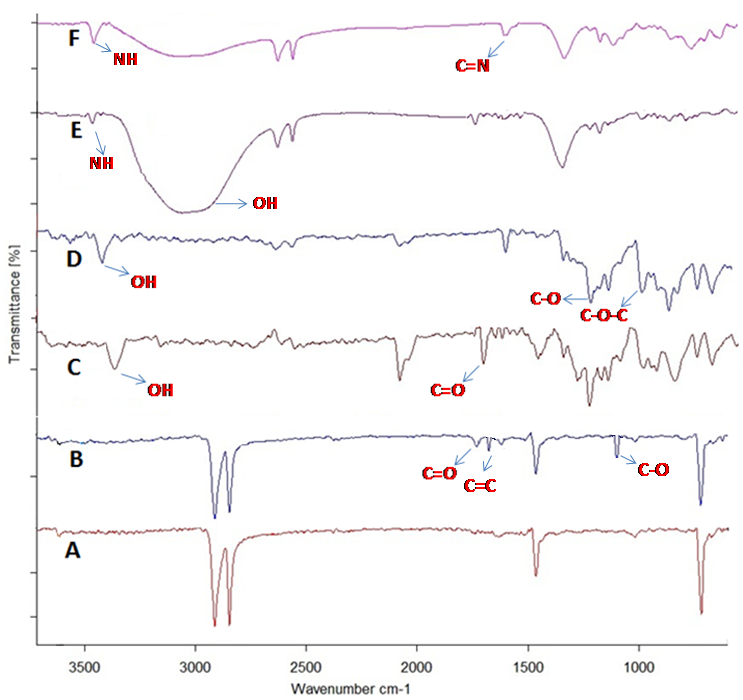
**

**Figure D:** FTIR spectra of (A) Non UV treated LLDPE, (B) UV treated LLDPE, (C) Non UV treated CC-LLDPE, (D) UV treated CC-LLDPE, (E) Non UV treated PCC-LLDPE and (F) UV treated PCC-LLDPE.

**
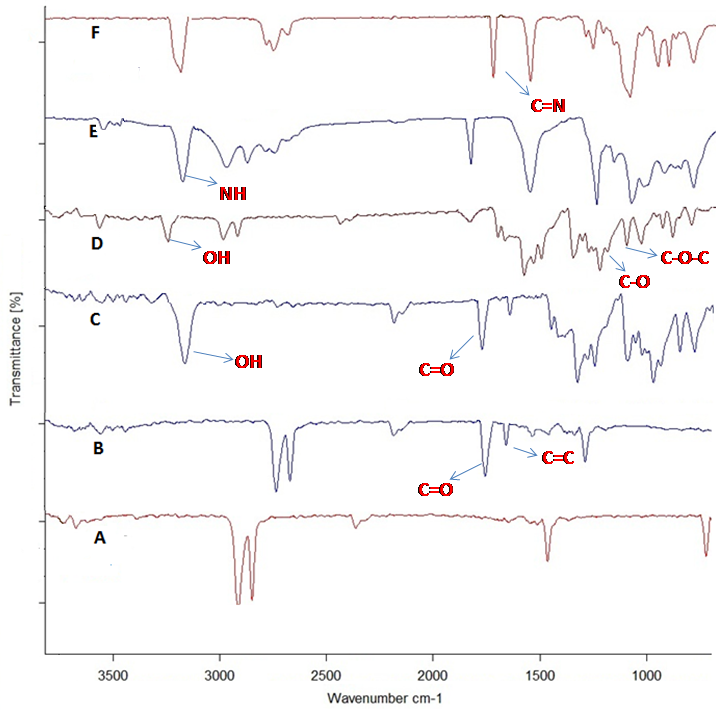
**

**Figure E: Organism hydrophobicity with bath assay**


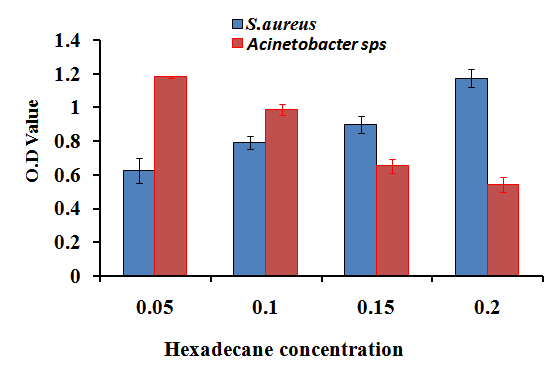


**Table A FTIR details**

| **POLYMER** | **C=O** | **C=C** | **C-O** | **C-O-C** | **C=N** | **NH** | **NO** | **OH** |
| --- | --- | --- | --- | --- | --- | --- | --- | --- |
| LDPE NON UV TREATED | _ | _ | _ | _ | _ | _ | _ | _ |
| LDPE UV TREATED | 1734 cm^-1^ | 1640 cm^-1^ | 1274 cm^-1^ | _ | _ | _ | _ | _ |
| LDPE+CC NON UV TREAT | 1743 cm^-1^ | 1626 cm^-1^ | _ | _ | _ | _ | _ | 3290 cm^-1^ |
| LDPE+CC UV TREATED | _ | _ | 1150 cm^-1^ | 1050 cm^-1^ | _ | _ | _ | 3167 cm^-1^ |
| LDPE+CC+PCC NON UV TREATED | 1724 cm^-1^ | _ | _ | _ | _ | 3245 cm^-1^ | ­_ | 3245 cm^-1^ |
| LDPE+CC+PCC UV TREATED | _ | _ | 1077 cm^-1^ | 1136 cm^-1^ | 1624 cm^-1^ | 3254 cm^-1^ | _ | 3254 cm^-1^ |
| PCL NON UV TREATED | 1721 cm^-1^ | _ | _ | _ | _ | 3425 cm^-1^ | _ |  |
| PCL UV TREATED | 1725 cm^-1^ | _ | 1146 cm^-1^ | _ | _ | _ | 1550 cm^-1^ |  |
| PCL+CC NON UV TREATED | 1730 cm^-1^ | _ | _ | _ | _ | 1596 cm^-1^ | _ | 3340 cm^-1^ |
| PCL+CC UV TREATED | 1724 cm^-1^ | _ | 1285 cm^-1^ | 1150 cm^-1^ | _ | _ | 1375 cm^-1^ | 3348 cm^-1^ |
| PCL+CC+PCC NON UV TREATED | 1720 cm^-1^ | _ | _ | _ | _ | 3244 cm^-1^ | _ | 3200 cm^-1^ |
| PCL+CC+PCC UV TREATED | 1721 cm^-1^ | _ | 1330 cm^-1^ | 1071 cm^-1^ | 1625 cm^-1^ | 3251 cm^-1^ | 1381 cm^-1^ | 3225 cm^-1^ |
| HDPE NON UV TREATED | _ | _ | _ | _ | _ | _ | _ | _ |
| HDPE UV TREATED | 1724 cm^-1^ | 1630 cm^-1^ | 1023 cm^-1^ | _ | _ | _ | _ | _ |
| HDPE+CC NON UV TREATED | 1739 cm^-1^ | 1626 cm^-1^ | _ |  | _ | _ | _ | 3345 cm^-1^ |
| HDPE+CC UV TREATED | _ | _ | 1285 cm^-1^ | 1114 cm^-1^ | _ | _ | _ | 3420 cm^-1^ |
| HDOE+CC+PCC NON UV TREATED | _ | _ | _ | _ | _ | _ | 3456 cm^-1^ | 3222 cm^-1^ |
| HDPE+CC+PCC UV TREATED | _ | _ | _ | _ | 1624 cm^-1^ | _ | 3420 cm^-1^ | 3295 cm^-1^ |
| LLDPE NON UV TREATED | _ | _ | _ | _ | _ | _ | _ | _ |
| LLDPE UV TREATED | 1732 cm^-1^ | 1634 cm^-1^ | _ | _ | _ | _ | _ | _ |
| LLDPE+CC NON UV TREATED | 1721 cm^-1^ | 1632 cm^-1^ | _ | _ | _ | _ | _ | 3226 cm^-1^ |
| LLDPE+CC UV TREATED | _ | 1626 cm^-1^ | 1300 cm^-1^ | 1123 cm^-1^ | _ | _ | _ | 3310 cm^-1^ |
| LLDPE+CC+PCC NON UV TREATED | _ | _ | _ | _ | _ | 3323 cm^-1^ | _ | 3421 cm^-1^ |
| LLDPE+CC+PCC UV TREATED | _ | _ | _ | _ | 1633 cm^-1^ | 3326 cm^-1^ | _ | 3245 cm^-1^ |
